# Reframing Sexual Health for Black Girls and Women in HIV/STI Prevention Work: Highlighting the Role of Identity and Interpersonal Relationships

**DOI:** 10.3390/ijerph182212088

**Published:** 2021-11-18

**Authors:** Ijeoma Opara, Jasmine A. Abrams, Kristina Cross, Ndidiamaka Amutah-Onukagha

**Affiliations:** 1 Yale School of Public Health, Yale University, New Haven, CT 06510, USA; 2 School of Public Health, Boston University, Boston, MA 02118, USA; abramsja@bu.edu; 3 School of Social Welfare, Stony Brook University, Stony Brook, NY 11794, USA; kristina.cross@stonybrook.edu; 4 Department of Public Health, School of Medicine, Tufts University, Boston, MA 02111, USA; Ndidiamaka.amutah_onukagha@tufts.edu

**Keywords:** Black girls, sexual health, sexual behavior, Black women

## Abstract

While Black girls and women are disproportionately impacted by sexual health disparities, there continues to be an overwhelming focus on individual risk behaviors within prevention initiatives, which offers a fragmented narrative of the multidimensional nature of risk and plausibly limits effectiveness of prevention programs and attenuates reductions in disparities. Because sexual health is experienced within an individual’s beliefs/values, interpersonal relationships, and behaviors and reflects larger social and cultural systems, it is important to critically examine common theories used to inform HIV/STI prevention interventions for Black women and girls. To fill this gap in the literature, we critique two commonly used theories in HIV/STI prevention interventions, namely the social cognitive theory and the theory of gender and power, by highlighting theoretical and practical strengths and weaknesses. We propose research implications that incorporate key strengths of the two theories while adding new concepts grounded in the intersectionality theory. The overall goal is to introduce a more comprehensive conceptual model that is reflective of and applicable to the multidimensional sexual experiences of Black girls and women within the evolving definition of sexual health and behavior.

## 1. Introduction

Sexual health refers to the social, psychological, interpersonal, and intrapersonal functions of sex as a core element of health across the life span from adolescence through to later adulthood [1]. Sexual health is often discussed in the literature as a paradigm relevant to the prevention of risk behaviors that increase disease infection and unwanted pregnancy. There has been an overwhelmingly focus on individual-level risk behaviors as a factor in high incidence rates of STIs among Black girls and high HIV rates among Black women [2]. The data is concerning, yet prevention programs have not fully addressed the factors that continue to place Black girls and women at risk, which may continue to widen the disparity gaps in their health outcomes. Sexuality is connected to almost every arena of daily life, yet it is discussed often through a negative, risk-perspective lens for Black girls and women as opposed to an empowering and liberating experience. Sexual health is multidimensional. It is experienced within an individual’s beliefs/values, relationships, and behaviors and reflects larger social and cultural systems. It is important for prevention programming to reframe the view of sexual health and behaviors among women and girls, specifically for Black girls and women. Because STI and HIV prevention research tends to place focus on individual risk behaviors as opposed to structural factors, such as systemic racism, sexism, stigma, medical mistrust, and homophobia, the full lived experiences of Black girls and women in the U.S. have not been fully taken into account in theoretical frameworks that ground the most commonly used HIV and STI prevention programs. There are major considerations that need to be understood that contribute to sexual health outcomes in Black girls and women. Theories used to develop interventions for Black girls and women should first highlight the historical context of how the relationship between sexual and romantic relationships involving Black girls and women are conceptualized, how stereotypes have impacted their views and expected behaviors, and the continued medical mistrust that is experienced by this group.

## 2. Historical Context of Enslaved Black Girls and Women

The sexuality of Black girls and women has been scrutinized and misunderstood from the beginning of institutionalized slavery up until the present time. Within a historical sociocultural perspective, the need to understand the impact of slavery on Black girls and women and its residual effects that contribute to stereotypes and beliefs placed on Black girls and women can be crucial in reframing sexual health outcomes. Black Americans have a unique sociocultural perspective as they have endured massive historical traumatic experiences of 400 years of slavery and post-Jim Crow era. Even present-day experiences of racism and discrimination, which have continued to penetrate into American culture, leave long-standing consequences that contribute to mental and physical health outcomes. Very few, if any, health behavior theories used in HIV and STI prevention research have attempted to link the connection between historical trauma and sexual health and the behavior among Black girls and women in the present day and how they are treated within relationships.

Enslaved Black girls and women were sexually exploited as slaves, brutally raped, and abused by White masters and other White males without legal repercussions. Enslaved Black girls and women were viewed as extremely profitable by White slave masters due to their ability to work in the fields as men [3]. However, when it came to sexual exploitation, their roles were exclusively based on their gender and were used for sexual purposes by their slave masters. Enslaved Black girls and women quickly learned to internalize this belief that their bodies were only good for sexual purposes and began to use sex to receive favor from their slave masters, such as better housing quarters, food choices, and treatment of their children [4,5,6]. The historical treatment of Black girls and women has contributed to the stereotypes that are placed on this group in the present day. Black girls and women are viewed as promiscuous, overly sexual, and sexually irresponsible in society [7]. Decades after slavery was abolished in 1865, Black people witnessed and experienced sexual exploitation, raping of women and girls without legal repercussions, and widespread attitudes and practices of racial oppression, humiliation, and discrimination, reminiscent of the traumatic ordeals they endured during slavery. The historical degradation of enslaved Africans served as a traumatic event; thus, the devaluation of women has been inherited through multiple generations. Due to this trauma, sexual pleasure and desire are often ignored within the context of Black girls and women, leaving Black girls and women to primarily seek sex to please their partners while unconsciously denying their power to engage in sexual activity in ways that would make them comfortable. This can leave Black girls and women in positions where they negate their needs and participate in health-compromising behaviors.

## 3. Medical Mistrust

In addition to mass genocide that occurred to enslaved Africans in the U.S., Black people as a whole also tend to mistrust medicine and research due to the history of dishonesty by medical professionals and researchers. As a construct, mistrust is the belief that an individual or entity is acting in opposition to one’s best interest or wellbeing. Research has examined this concept and found that among Black women, medical mistrust or mistrust of healthcare providers, healthcare institutions, and government in relation to healthcare may impact the ability to have sexual health conversations with providers [8] as well as HIV testing [9] and PrEP uptake [10,11]. Sexual behavior and health research rarely takes into account the mistrust of medical providers and how such beliefs can be passed down intergenerationally. In addition, medical providers continue to grapple with racist and sexist practices that are harmful to Black girls and women due to the inherent bias and stereotypes placed on this group, thereby leaving Black girls and women unwilling to trust their medical providers and rely on other resources. The sociohistorical context of Black people in the United States has allowed scholars to examine the ways intergenerational trauma can impact the attitudes, beliefs, values, and behaviors that inform the identity of Black girls and women. Although some of these behaviors may serve as a catalyst for survival in a society that continues to view Black girls and women through deficit lens while dismissing their inherited generational trauma, individual-level behaviors need to be taken into account within a larger context.

In this article, we highlight and critique theories that have been primarily seen as foundational theories for HIV/STI prevention intervention work for Black girls and women. While we are not attempting to discredit or critique these well-established theories, we are calling for development of a new theoretical model that is applicable to our current experiences in the evolving definition of sexual health and behavior. There has been more awareness of the various intersections that affect the health of Black girls and women, yet a fully comprehensive sexual health model has not been created specifically to address such factors. Therefore, we provide research implications for the development of an intersectional model of sexual health for Black girls and women that can be utilized to include various aspects of the common foundational theories while interweaving new intersectional concepts.

## 4. Theories Used in Sexual Behavior Research: Social Cognitive Theory and Theory of Gender and Power

After reviewing prevention interventions approved by the Center for Disease Control and Prevention (CDC) Compendium of Evidence-Based Interventions and Best Practices for HIV Prevention specifically for Black girls and women, nine interventions were found. Six out of the nine interventions were grounded in the social cognitive theory (over 50%), followed by three interventions that used the theory of gender and power (see Supplementary Materials). The use of a theory in an intervention that attempts to explain human behavior is predicated on the extent to which empirical data support its key tenets. However, several authors have emphasized that theories, including psychological and human behavior theories, should not be unchallenged or considered complete if empirical evidence suggests the need for theoretical amendment [12,13].

**Social cognitive theory:** Social cognitive theory is a common theory that has been used in HIV risk behavior programs for Black girls and women, such as SISTA and SIHLE [14] and PHA: Promoting Health Among Teens [15]. SCT views behavior and behavioral change as a social process influenced by interaction with people and environments [16,17]. The theory assumes that behavior is often learned and posits that learning occurs in a social context with a dynamic and reciprocal interactions that influence behavior. The theory heavily relies on how an individual is influenced by relationships, and its emphasis is on social influence and its role on social reinforcement. SCT considers the unique way in which individuals acquire behaviors and knowledge and the social and immediate environments in which individuals perform the behavior [16,17]. The theory considers personal past experiences that factor into whether behavioral action will occur. These past experiences can impact expectation and expectancies, all of which shape whether a person will engage in a particular behavior and influence the reason why the person engages in that behavior. SCT comprises four concepts:

1. *Reciprocal determinism:* A person’s behavior is shaped by environment, immediate peers, and behaviors seen in others.
2. *Outcome expectations*: A person’s behavior is motivated by what they expect the particular outcome to be. A person will only change their behavior if they expect that a particular consequence will occur.
3. *Self-efficacy:* A person will change behavior if they believe they have the power to engage in that behavior in other settings or situations.
4. *Observational learning:* This refers to peer modeling and the importance of an individual viewing the behavior of someone else in order to execute the behavior.

**Critique:** A key theoretical mediator within SCT links self-efficacy beliefs to how behavioral accomplishments correspond to goal setting. Within the context of sexual behavior and health for Black girls and women and other groups, the goal is to engage in less “risky” behaviors that may lead to disease infection. In addition, SCT focuses heavily on individual behaviors as key to mitigating outcomes, which leads prevention interventions to encourage participants to reduce individual-level behaviors as they may result in negative consequences. According to Bandura [16,18], people envision certain positive outcomes resulting directly from their behaviors only if they have the perceived capabilities to perform those behaviors in the first instance. Additionally, if individuals do not believe they have the capability needed to perform a certain behavior, they will not envision positive outcomes deriving from that behavior. Similar models, such as the health belief model, prescribe to this logic as well. This belief, however, demonstrates that self-efficacy has to occur before outcome expectations. However, a critique of SCT provided by Williams and Rhodes [19] points to expectations about future outcomes as being a causal factor of self-efficacy beliefs. For example, experimental studies have shown that when people are provided with monetary incentives (to reduce sexual behavior, e.g., SISTA) or asked to envision the negative consequences of a health-compromising behavior (i.e., HIV and STIs), their self-efficacy beliefs tend to improve. In two studies aimed at influencing behavioral change and improving self-efficacy, the outcome expectations were targeted (via incentives tied to the behavior and envisioned health consequences). This was done in order to achieve certain outcomes using theoretical sources of self-efficacy (as per Bandura, 1977a, 1997) [16,18], and results showed an improvement in self-efficacy beliefs. These findings suggest a potential need to reconceptualize SCT, with outcome expectations positioned as an additional “source” of self-efficacy beliefs within the context. Moreover, outcome expectations are often viewed through disease prevention lens as opposed to highlighting the benefits of sex on mental health, intimacy, and liberation for Black girls and women [20]. In sum, it is plausible to suggest, based on emergent empirical evidence, that SCT can be reconceptualized within the context of sexual behavior to position outcome expectancies as both a source and consequence of self-efficacy beliefs and to reframe outcomes. Regarding the observational learning factor of SCT, Black girls and women are forced to navigate through environments that reinforce individual-level behaviors that can increase risk of disease infection or unplanned pregnancy. However, little discussion of how to model safe sexual behavior and rewards given to those who engage in safe sex, such as pleasure, is rarely discussed in prevention research.

**Theory of gender and power:** The theory of gender and power (TGP) acknowledges that there are power imbalances in heterosexual relationships, which leave girls and women at risk [21]. TGP provides an explanation to such imbalances as economically driven and embedded in societal norms. Such inequality becomes embedded in society where women feel they are “less than” men and influences decision-making that lacks in protecting their own self-interest. According to TGP, such views place women at increased risk of STIs and HIV/AIDS. TGP explains that adolescent females and women of color are most at risk of engaging in risky sexual behaviors, which increase their likelihood of contracting STIs or HIV due to their vulnerable status [22]. In the context of Black girls and women, TGP provides a rationale for the negative consequences that are associated with sexual activity in heterosexual relationships. A few factors that have been discussed include the relationship dynamic between the two genders, the perceived sex ratio imbalance, and the balance of trust and power between the two individuals [23,24]. Studies have found that the perceived power difference between the two sexes due to the assumed gender ratio imbalance can impact whether Black women engage in behaviors that put their sexual health as a priority. One study [24] found that Black women perceived the lack of desirable male partners and the perceived gender ratio imbalance in Black communities as potential factors that limited women’s ability to negotiate monogamy in their relationships as well as difficulty in negotiating condom usage. The potential consequences of this perceived gender ratio imbalance include the possibility of Black men feeling able to have multiple sexual partners and of Black women adapting to the condom preferences of their male partners. Given this assumed power imbalance between the sexes, Black women may feel less inclined to negotiate condom use or safer sex practices under the assumption that she may lose her partner if she does so [25].

**Critique:** Although TGP discusses the power imbalance between men and women in heterosexual relationships, it does not account for the historical degradation of Black women and girls that has contributed to the HIV/AIDS epidemic. Such a view on Black girls and women is embedded in society and unconsciously internalized by girls and women. Black girls and women have the unfortunate role of having to negate negative images of themselves, which unconsciously shapes their sexual identity and behaviors. In addition, the theory does not take into account how the historical degradation of Black girls and women has impacted Black heterosexual romantic relationships and the view that Black males may have towards Black female partners. One study found that Black girls tend to be seen as undesirable by Black boys, which then may lead to negative views of themselves and result in them engaging in unhealthy behaviors [26]. Another study found that there tends to be an imbalance in power among Black heterosexual couples that is predominantly shaped by mistrust of Black men’s inability to be safe and supporting romantic partners [27].

Furthermore, the theory lacks in discussion of how power imbalances can arise in same-sex relationships [28]. The assumption that heterosexual women are most at risk can be attributed to the fact that 87% of HIV transmission among women is through heterosexual sex. However, discussion of women and girls who are in bisexual relationships or same-sex relationships are often left out of the literature. Heterosexual behaviors are often discussed throughout prevention interventions that are targeted towards Black girls and women, which contributes to the idea that only heterosexual women are at risk of HIV/AIDS and STIs. This can be a concerning assumption as the majority of Black lesbians and women who have sex with women have had sex with a male partner at some point in their life [29,30,31,32]. In addition, Black lesbians and women who have sex with women may be less likely than heterosexual women and women with only male sexual partners, respectively, to have received contraception due to perceptions made by their healthcare provider due to their sexual orientation [33,34] and less likely to be screened for STIs and HIV [35].

## 5. Theoretical Considerations Moving Forward: Intersectionality and Sexual Behavior in Black Girls and Women

While there are certainly a number of potential factors that intersect to contribute to poor sexual health outcomes for Black girls and women, one aspect that requires more research is the role of sexual stereotypes of Black girls and women. Black women and girls continue to be inundated with negative images and expectations for how they should behave sexually. Black girls and women have often found themselves encountering predominantly four stereotypical roles that have been derived from the institution of slavery. A study examining the relationship between endorsement of stereotypical roles and sexual behavior that is viewed as “risky” amongst Black female college students found that there was a correlation between three of the stereotypical roles and the participants engaging in risky sexual behavior [36]. Important theoretical considerations are needed that acknowledge the positionality of Black girls and women and their various identities that contribute to their perception and their oppression.

## 6. The Use of Intersectionality Theory in an Integrated Framework

Researchers often view salient contextual variables, such as race, ethnicity, gender, sexual orientation, socioeconomic status/class, and ability, as separate sociocultural demographic variables that rarely influence one another. Yet, intersectionality theorists contend that contextual variables intersect and influence one another, resulting in specific outcomes [37]. Although intersectionality has been conceptualized in various ways, researchers have suggested that an individual’s multiple identities interact and intersect to shape personal experiences [37] and at times form “intersecting oppressions … that work together to produce injustice” [38]. Within the sexual health context, in order to accurately discuss the experiences of Black girls and women, it would be a disservice to fail to incorporate a theory that specifically acknowledges the social locations that intersect to create multiplicative oppressive experiences for Black girls and women. Intersectionality [37] describes how multiple forms of oppression can affect individuals, couples, and families due to race, gender, and class and even lead to barriers to forming healthy relationships, produce unbalanced power dynamics in romantic partnerships, and consequently lead to poor sexual health outcomes for Black girls and women in vulnerable positions [39]. Class differences tend to be overshadowed by race and gender inequities for Black girls women with regard to safe sex practices [24], and few studies have understood the sexual decision-making and processes of upper- and middle-class Black girls and women.

It is critical then that the tensions Black girls and women witness, the oppression they experience, and the structural contexts that continue to marginalize them be made visible to gain a clearer understanding of the circumstances that place them at risk in order to support Black girls and women in achieving positive sexual health outcomes. In spite of recent acknowledgements in family science literature that highlight the influence of gender in intrapersonal and interpersonal interactions (e.g., microprocesses), a gap persists in conducting intersectional analyses on how Black girls (and women) and their families navigate changing systemic sociopolitical and economic obstacles and barriers [40,41]. Incorporating intersectional concepts in HIV prevention work goes beyond highlighting categories and identities that Black girls and women belong to and extends to providing appropriate solutions to enhance foundation theoretical constructs. Such an approach allows the field to be more responsive and sensitive to the various experiences of Black girls and women.

## 7. Increasing Sexual Self-Efficacy in Black Girls and Women

Bandura’s concept of self-efficacy as a part of social cognitive theory is described as one’s belief in their ability and power to impact events in their lives through their behavior, also known as perceived self-efficacy [42]. Within the intersectional framework of sexual behavior and health, observed factors of sexual self-efficacy that should be highlighted and normalized in prevention are confidence and preference in using condoms during intercourse, sexual communication, and sexual negotiation. Sexual self-efficacy is defined in the literature as the belief in one’s ability to navigate a sexual context appropriately, advocate for desired psychological and physical pleasure within sexual experiences, refuse unwanted sexual activities, and incorporate the discussion and use of contraceptive and sexual protection methods [43,44,45]. One study of young adults found that perceptions about how condom use reduced sexual pleasure was more strongly associated with not using condoms than any sociodemographic information collected within that study [46]. Additionally, a study of Black women with low income found that positive attitudes of condom use was associated with more condoms use [47]. Among women, perception of one’s sexual self-efficacy was a greater predictor of risky sexual behavior then knowledge about safe sex practices [45].

Greater perceived sexual self-efficacy within Black girls and women overall increases condom use, sex refusal, and avoidance of harmful sexual risk behaviors [48] and consequently decreases the number of Black women acquiring HIV and STI as well as unwanted pregnancies associated with these behaviors. Various interpersonal, societal, and cultural factors (i.e., sexual scripts, self-esteem, sexual self-confidence, and emotional safety) have shown to influence sexual decision-making and perceived sexual self-efficacy [45,49,50,51]. Gause [52] found poorer self-worth and self-love were associated with lower partner communication self-efficacy, greater fears related to condom negotiation, greater endorsement of peer norms for risky sexual behavior, and lower partner trust and low sexual communication with partners among Black women.

It is important to note that sexual self-efficacy can be difficult to achieve as Black girls and women must navigate between the double standard in which girls and women are judged negatively for engaging in the same sexual behavior that boys and men engage in [53]. Another study [54] which tested sexual double standards for Black females in the United States found that nonassertive sexual scripts, where Black females do not feel confident in taking charge of decisions surrounding sex with their romantic partners, limited Black female’s agency in planning for safe sex. Redmond and Lewis [55] found that having high sexual negotiation skills and a sexual partner who approved of condom use were significant predictors for high perceived sexual self-efficacy among Black women. More female adolescents intended to use condoms if their sexual partners approved of condom use compared to their male counterparts. Though perceived self-efficacy is an efficient protective tool to assist positive sexual decision-making, it can be improved or worsened by social, cultural, or environmental factors [42,55]. The intersections of Blackness, girlhood, and womanhood within American society remains a unique experience that requires more scientific research to inform development of treatment modalities and interventions. While prevention interventions for Black girls and women should highlight and encourage sexual self-efficacy, providing the same training to Black boys and men would be needed to combat stereotypes, sexual coercion, and views of self that Black females tend to grapple with.

## 8. Sexual Agency: Balancing Power Dynamics in Romantic Relationships

Most of the research surrounding Black female sexual activity is focused on risky behaviors that can lead to unplanned pregnancies and transmission of HIV and other sexually transmitted infections [20,56]. The perspective of Black girls and women on sexual behavior is vital to this body of literature and for prevention overall as it is essential to promote safe sexual practices and to holistically address Black women’s sexuality. Scholars have argued that Black female sexual scripts are rooted in heteronormative, patriarchal, racial, and classist structural social factors [57]. However, the current literature on sexual subjectivity and sexual agency dismisses these in favor of sociohistorical factors more associated with wealthy White women and individual sexual decision-making [58,59,60]. The perspectives of Black women about sexuality and sexual behaviors through the lens of the historical gendered racial inequities that have impacted the sexuality of Black girls and women are essential to address sexuality within Black womanhood and influence sexual scripts.

Sexual scripts are described as socially shared meanings of sexual behavior that outline the sequence of events in well-known situations, influence personal sexual behavior, and develop attitudes about one’s sexual self [61,62,63]. Sexual scripts associated with Black girls and women are the Diva, Gold Digger, Freak, Dykes, Gangster Bitch, Earth Mother, Sister Savior, Baby Mama, Jezebel, and Mammy [57,63]. A qualitative study exploring the motivations of Black women and Latinas who engage in unprotected sex with male partners found that maintaining hope, sensuality, strategic gain, and stability with the male partner were their primary goals for having unprotected sex with their partners [64]. In low-power sexual scripts, known risks of HIV were overpowered by an obligation to satisfy a male partner and accept cheating, while high-power sexual scripts highlighted Black women’s awareness of themselves as worthy of self-care [64]. Studies show that the root of this motivation can be attributed to racial and gendered inequities that exist in society. In a study that included a sample of both Black and White women aged 20 to 45 years, Black women were more likely to start a relationship due to economic considerations and have transactional sex with someone who was not a regular partner than White women [65]. Another study looking at a sample of Black women found that motivations for having sex with multiple male partners or having extra-relational sexual encounters included economic advancement and stability and emotional benefits [66]. Together, the findings in this area highlight a gap in the literature of a well-rounded understanding of how Black girls and women view the activity of sex and sexual behavior and the agency they may lack in their relationships with men. Much of the existing literature on Black girls and women point to underlying motivations for sexual activity in an attempt to explain Black women’s engagement in risky sexual behavior but do not address the attitudes of Black girls and women towards sexual activity.

## 9. Creating Space in Sexual Health Programming for Sexual and Gender Minority Black Girls and Women

Within the sexual health literature, sexual orientation is rarely discussed as being on a continuum among Black girls and women. Because sexual orientation is a construct that comprises multiple factors, such as sexual attraction, sexual identity, and sexual behavior, it can have the potential to influence health differently [67]. As clinicians and researchers may have a dichotomous view of sexuality (e.g., heterosexual or homosexual) without considering the fluidity of sexuality, this can contribute to inaccurate misconceptions, shame, and risk among Black girls and women, leaving them at risk of poor sexual health outcomes. Sexual health experiences of Black sexual minority girls and women are affected by multiple forms of discrimination, including sexism, racism, classism, and heterosexism, and may differ from those of their White counterparts. Therefore, most studies on sexual health among Black girls and women may not be generalizable to sexual minority Black girls and women. The literature on Black women’s sexuality and sexual behavior focuses on monoheterosexuality and those with low socioeconomic status [68]. This has created a monolithic view of Black girls and women and has overshadowed the potentially unique methods of maintaining or achieving sexual health present in the interactions of Black women who have sex with women and the impact of class differences on Black women.

Within lesbian culture, there exist “gendered scripts”, such as butch, stud, and femme. Researchers contest that these stereotypes are strictly based on heterosexual norms, with butch and stud as the male role and the femme as the more feminine role within romantic relationships [68]. A focus group that included a sample of Black women who identified as lesbians [69] found that while the participants acknowledged the dominant sexual discourses of choosing polar opposite identities on a feminine–masculine continuum, the actual expression of sexuality did allow for gender blending. The study found that although studs and femmes appear to operate within heterosexual roles for women and men, femmes, who would be expected to be passive and nonassertive in playing the woman’s role in sexual engagement, fully expected that sexual acts ended with their sexual climax [69]. Even more critical are Black girls and women who identify as gender minorities. Black transgender girls and women are often ignored within HIV prevention programming due to the absence of transgender-specific guidelines [70]. Additional risk factors that may uniquely affect transgender girls and women involve nonprescription hormone therapy, which may increase HIV risk. In addition, experiences related to transphobia may affect the way Black transgender girls and women interact with their romantic partners and may result in intimate partner violence and sexual assault, which are risk factors for HIV [71]. This is concerning as Black transgender women as an example are disproportionally diagnosed with HIV [72,73]. Yet, very little is known about sexual scripts and gendered norms that Black transgender girls and women may adopt that can manifest within their romantic relationships. Within theories that highlight patriarchal dominance and emphasize the role on gender, such as the theory of gender and power, it is crucial that more attention is placed on how such scripts arise within Black lesbian and same-sex romantic relationships and also among transgender girls and women.

## 10. Prioritizing Sex Positivity and Pleasure in Prevention Programming and Moving Away from Guilt

Historically, sex-positive narratives were constructed in response to sex-negative dominant, pathology-based lenses. They challenged theories of deviant sexuality; proposed that sexual expressions are healthy, pleasurable, and essential aspects of human development; criticized paradigms of sexuality wherein female sexuality is defined in relation to men; and questioned sexual repression and sexual binaries [74]. Sexual positivity can include sexual satisfaction, sexual self-esteem, sexual pleasure, and sexual efficacy and can positively influence sexual, mental, and physical health [75]. McClelland et al. [76] poses intimate justice as a theoretical framework that encourages researchers to questions how racist and sexist stereotypes, stigma, and identity impact how individuals process pleasure expectancies and barriers that hinder a positive view on sex (e.g., sexual guilt). Within the context of Black girls and women, various factors play a role in how sexual guilt arises, such as religion, familial structure, racism, sexism, and classism, which can therefore lead to the experience of having sex as one filled with shame and secrecy and deemed unenjoyable. While one of the most common reasons for engaging in sexual activity is for the expectation of physical pleasure [77], heterosexual Black girls and women often view the act of having sex as an activity to pleasure their male partners as priority over their own pleasure [78].

The concept of positivity in relation to wellbeing and public health outcomes is not isolated to sex and sexual behaviors. Gallagher and Updegraff [79] conceptualized the concept of gain-framed and loss-framed messaging. Gain-framed messaging emphasizes the benefits of engaging in a specific behavior, while loss-framed messaging focuses on the consequence of failing to engage in a specific behavior [79]. Brickman and Willoughby [80] found that gain-framed messages were preferred over loss-framed messages, with participants rating sex-positive messages as more believable and persuasive. Within health prevention messaging for Black girls and women, the parent–child relationship is often discussed as the most beneficial in reducing sexually risky behavior among Black girls [81]. However, in a few Black mother–daughter dyad studies, the findings consistently revealed that Black mothers continue to speak to their daughters less frequently about sex and report great discomfort when doing so [82,83]. While sexual guilt in adolescent girls is often attributed to familial structure and family’s views on sex, without interventions aimed at teaching Black girls and women about the importance of sex and removing the stigma of discussing sex, prevention research will continue to miss important opportunities to participate in positive role modeling and learned behaviors that exhibit healthier interactions and can reduce sexual health disparities.

Most of the research on Black girl’s sexuality and sexual health is skewed towards reducing risky behaviors and has less emphasis on healthy and normal aspects of sexuality and sexual behaviors, including sex positivity. After reviewing 245 articles to determine which topics surrounding sexualities were published most for Black women, Hargons et al. [20] found only 6.5% of the articles discussed sex positivity. A lack of intentionally inclusive sex-positive research, education, and public health promotion focusing on intersecting identities of Black womanhood across the lifespan can lead to a decrease in sexual health and overall wellbeing.

Family environment is often seen as a crucial part of sexual development and sexual health within the Black community [84,85]. Black girls often bear the brunt of negative messaging compared to Black boys in their family [84,86]. While negative messaging may prove effective in delaying sexual debut, Black girls may be more likely to feel guilt, less likely to have pleasurable sexual experiences, and may not advocate for their sexual desires and pleasures when they decide to engage in sex [87]. Sexual guilt can result in adverse health outcomes throughout their lifetime [87]. Additionally, it may lead girls to gain additional messaging from other sources, such as primary friends or the media [86], which may be unreliable or promote risky behaviors [88]. Together, sex-positive and gain-framed messaging within family- or community-based prevention programming can be a more effective method of promoting preventative practices, sexual safety, and consequently sexual wellbeing and reducing the stigma attached to sex for Black girls and women.

## 11. Conclusions

While social cognitive theory and theory of gender and power remain very popular frameworks used in prevention programming, intersectionality provides an additional consideration to further examine and understand the role of multiple identities and categories, along with the interactions of individuals and groups on which public policies, practices, and social institutions have a direct impact. While Black girls and women encompass various identities that this article may not have fully addressed (e.g., religion and disability), we encourage researchers to be aware of an intersectional framework in order to achieve sustainable results. Through an examination of the interactions between social identity and social institutions, intersectionality theory highlights how power and privilege are negotiated at the individual, community, and structural levels. The intersectional integrated framework we propose includes four components that are grounded in social cognitive theory, theory of gender and power, and intersectionality theory: (1) increasing sexual self-efficacy in Black girls and women in order to normalize confidence and preference for using safe sex practices through improved communication and negotiation around sex; (2) encouraging sexual agency in order to balance out power dynamics in romantic relationships that impact Black girls and women due to gender norms and challenge the patriarchal and racist structure through liberation and empowerment of Black girls and women; (3) creating space in sexual health programming for sexual and gender minority girls and women in order to acknowledge that power dynamics within romantic relationships may arise in same-sex relationships, address the lack of attention placed on Black transgender girls and women, challenge heteronormativity due to gendered scripts, and encourage the use of tools for girls and women to be aware of and combat; and (4) prioritizing sex positivity and pleasure in prevention programming and moving away from guilt in order to encourage positive discussions around sex, move away from shame, and enhance sexual satisfaction, sexual self-esteem, sexual pleasure, and sexual efficacy.

It is essential for Black girls and women to achieve liberation from the oppression that they have experienced around sexual behavior in order for effective and positive sexual health work to begin. It is essential to not only work with Black girls and women but to also educate their potential partners, families, and communities in which they are housed for there to be a change in the narrative around sexual health for Black girls and women.

## Supplementary Materials

The following are available online at https://www.mdpi.com/article/10.3390/ijerph182212088/s1.
