# Supplementary material for: Reframing Sexual Health for Black Girls and Women in HIV/STI Prevention Work: Highlighting the Role of Identity and Interpersonal Relationships
Source: Int J Environ Res Public Health. 2021 Nov 18;18(22):12088. doi: 10.3390/ijerph182212088 (PMC8621381; doi:10.3390/ijerph182212088)
Supplement: Supplementary file 1 [file ijerph-18-12088-s001.zip › ijerph-1442894-supple.pdf]

Name of Intervention	Age Group of Population	Theory of Intervention	Goals of Intervention	Referecnce
Female and Culturally Specific Negotiation	18 years of age or older	Theory of Gender and Power Theory of Planned Behavior, Theory of Reasoned Actions, Social Cognitive theory, Transtheoretial Model of Change	Increased condom use with steady partner, reduce number of times or percent of times having sex with paying partner, reduced trading sex for drugs or money, reduce number of drug injections	Sterk, C. E., Theall, K. P., Elifson, K. W., & Kidder, D. (2003). HIV risk reduction among African-American women who inject drugs: A randomized controlled trial.*AIDS and Behavior, 7*,73-86.
Healthy Love	18 to 69 years old	Health Belief Model, Social Cognitive Theory, Transtheoretical Model	Increase consistent condom use and other latex barriers, reduce unprotected sex with male partners, increase sexual abstinence, promote HIV testing and receipt of test results	Diallo, D. D., Moore, T. W., Ngalame, P. M., White, L. D., Herbst, J. H., & Painter, T. M. (2010). Efficacy of a single-session HIV prevention intervention for black women: A group randomized controlled trial.*AIDS and Behavior, 14*, 518-529.
HORIZONS	15 to 21 years old	Social Cognitive Theory, Theory of Gender and Power	Reduce sexually transmitted diseases, Increase condom use, increase communication with male partners about safer sex and STDs	DiClemente, R. J., Wingood, G. M., Rose, E. S., Sales, J. M., Lang, D. L., Caliendo, A. M., Hardin, J. W., & Crosby, R. A. (2009). Efficacy of sexually transmitted disease/human immunodeficiency virus sexual risk-reduction intervention for African American adolescent females seeking sexual health services: A randomized controlled trial.*Archives of Pediatrics and Adolescent Medicine, 163*, 1112-1121.
Project IMAGE	No age limit	AIDS Risk Reduction Model, Motivational Interviewing	Increase condom use, reduce STI incidence, increase HIV/STI awareness, increase HIV/STI risk perceptions, enhance communication skills, reduce instances of abuse, reduce unintended pregnancy	Shain, R. N., Piper, J. M., Newton, E. R., Perdue, S. T., Ramos, R., Champion, J. D., & Guerra, F. A. (1999). A randomized, controlled trial of a behavioral intervention to prevent sexually transmitted disease among minority women.*New England Journal of Medicine, 340*, 93–100.
Sistering, Informing, Healing, Living, and Empowering (SiHLE)	14 to 18 years old	Social Cognitive Theory, Theory of Gender and Power	Reduce sexual risk behaviors, reduce sexually transmitted diseases (STDs) and pregnancy, enhance skills and mediators of HIV preventative behaviors	DiClemente, R. J., Wingood, G. M., Harrington, K. F., Lang, D. L., Davies, S. L., Hook, E. W., 3rd., Oh, M., K., Crosby, R. A., Hertzberg, V. S., Gordon, A. B., Hardin, J. W., Parker, S., & Robillard, A. (2004). Efficacy of an HIV prevention intervention for African American adolescent girls: A randomized controlled trial.*JAMA Journal of the American Medical Association, 292*, 171–179.
Project SAFE	15 to 45 years old	AIDS Risk Reduction Model	Reduce new chlamydia and gonorrhea infections, reduce risky sex behaviors	Shain, R. N., Piper, J. M., Newton, E. R., Perdue, S. T., Ramos, R., Champion, J. D., & Guerra, F. A. (1999). A randomized, controlled trial of a behavioral intervention to prevent sexually transmitted disease among minority women.*New England Journal of Medicine, 340*, 93–100.
Sister-to-Sister	18 to 45 years old	Social Cognitive Theory	Eliminate or reduce sex risk behaviors, prevent new STD infections	Jemmott, L. S., Jemmott, J. B., III, & O'Leary, A. (2007). Effects on sexual risk behavior and STD rate of brief HIV/STD prevention interventions for African American women in primary care settings.*American Journal of Public Health, 97*, 1034-1040.
Women's Co-Op	At least 18 years of age	African American Feminism, Empowerment Theory	Reduce sex risk behaviors and drug use, increase employment and housing status	Wechsberg, W. M., Lam, W. K., Zule, W. A., & Bobashev, G. (2004). Efficacy of a woman-focused intervention to reduce HIV risk and increase self-sufficiency among African American crack abusers.*American Journal of Public Health, 94*, 1165–1173.
Sisters Saving Sisters	12 to 19 years old	Theory of Reasoned Behavior, Theory of Planned Behavior, Social Cognitive Theory	Eliminate or reduce unprotected sexual intercourse and number of sex partners, prevent new STD infections	Jemmott, J. B., 3rd., Jemmott, L. S., Braverman, P. K., & Fong, G. T. (2005). HIV/STD risk reduction interventions for African American and Latino adolescent girls at an adolescent medicine clinic: A randomized control trial.*Archives of Pediatric and Adolescent Medicine, 159*, 440-449.
